# Effects of Repeated Ethanol Exposures on NMDA Receptor Expression and Locomotor Sensitization in Mice Expressing Ethanol Resistant NMDA Receptors

**DOI:** 10.3389/fnins.2017.00084

**Published:** 2017-02-21

**Authors:** Carolina R. den Hartog, Meghin Gilstrap, Bethany Eaton, Daniel H. Lench, Patrick J. Mulholland, Gregg. E. Homanics, John J. Woodward

**Affiliations:** ^1^Department of Neuroscience, Medical University of South CarolinaCharleston, SC, USA; ^2^Department of Anesthesiology, University of PittsburghPittsburgh, PA, USA

**Keywords:** western blot, OFC, PFC, BLA, hippocampus, accumbens, striatum

## Abstract

Evidence from a large number of preclinical studies suggests that chronic exposure to drugs of abuse, such as psychostimulants or ethanol induces changes in glutamatergic transmission in key brain areas associated with reward and control of behavior. These changes include alterations in the expression of ionotropic glutamate receptors including N-methyl-D-aspartate receptors (NMDAR) that are important for regulating neuronal activity and synaptic plasticity. NMDA receptors are inhibited by ethanol and reductions in NMDA-mediated signaling are thought to trigger homestatic responses that limit ethanol's effects on glutamatergic transmission. Following repeated exposures to ethanol, these homeostatic responses may become unstable leading to an altered glutamatergic state that contributes to the escalations in drinking and cognitive deficits observed in alcohol-dependent subjects. An important unanswered question is whether ethanol-induced changes in NMDAR expression are modulated by the intrinsic sensitivity of the receptor to ethanol. In this study, we examined the effects of ethanol on NMDAR subunit expression in cortical (orbitofrontal, medial prefrontal), striatal (dorsal and ventral striatum) and limbic (dorsal hippocampus, basolateral amygdala) areas in mice genetically modified to express ethanol-resistant receptors (F639A mice). These mice have been previously shown to drink more ethanol than their wild-type counterparts and have altered behavioral responses to certain actions of ethanol. Following long-term voluntary drinking, F639A mice showed elevations in GluN2A but not GluN1 or GluN2B expression as compared to wild-type mice. Mice treated with repeated injections with ethanol (2–3.5 g/kg; i.p.) showed changes in NMDAR expression that varied in a complex manner with genotype, brain region, subunit type and exposure protocol all contributing to the observed response. F639A mice, but not wild-type mice, showed enhanced motor activity following repeated ethanol injections and this was associated with differences in NMDAR subunit expression across brain regions thought to be involved in drug sensitization. Overall, while the results of the study suggest that NMDARs with reduced sensitivity to ethanol favor the development of locomotor sensitization, they also show that intrinsic ethanol sensitivity is not the sole determinant underlying changes in NMDAR expression following repeated exposures to ethanol.

## Introduction

Following repeated episodes of ethanol consumption, neuroadaptive changes in brain function arise that are believed to play an important role in the development of tolerance and physical dependence to ethanol. Ethanol's long-lasting effects on behavior have been linked to alterations in glutamatergic signaling that are similar to those involved in activity-dependent changes in synaptic plasticity. These long-lasting changes in glutamate neurotransmission may underlie the transition from initial alcohol consumption to uncontrolled and compulsive drinking (Gass and Olive, 2008; Szumlinski and Woodward, 2014).

Studies have shown that adaptations in glutamatergic signaling following exposure to ethanol include changes in the expression or localization of N-Methyl-D-Aspartate receptor (NMDAR) subunits (Follesa and Ticku, 1996; Snell et al., 1996; Hu and Ticku, 1997; Kalluri et al., 1998; Carpenter-Hyland et al., 2004; Wang et al., 2010; Kroener et al., 2012). Alterations in NMDAR subunit expression and function are thought to be an important component that underlies the increased excitability and neurotoxicity of neurons often observed during ethanol withdrawal (Hendricson et al., 2007; Nimitvilai et al., 2016) and may contribute to future drinking (Vengeliene et al., 2008). Although it is widely assumed that ethanol-induced changes in NMDAR expression and function reflect a homestatic up-regulation in response to receptor inhibition, this has not been directly tested and it is possible that these changes involve actions of ethanol on other cellular signaling processes.

To address this question, we utilized a novel mouse strain previously developed in our laboratory that expresses ethanol-resistant NMDA receptors. These mice were generated by codon replacement that changes a phenylalanine (F) at position 639 in the TM3 domain of the GluN1 subunit to an alanine (A) (den Hartog et al., 2013). Expressing GluN1(F639A) with various wild-type GluN2 subunits in oocytes (Ronald et al., 2001) and HEK293 cells (Smothers and Woodward, 2006, 2016) significantly reduces the sensitivity of these receptors to ethanol. NMDA EPSCs recorded from GluN1(F639A) mice are also markedly less sensitive to ethanol than those from wild-type controls and mutant mice exhibit changes in ethanol-induced locomotor activity, anxiolysis, motor impairment along with altered patterns of ethanol consumption as compared to their wild-type counterparts (den Hartog et al., 2013). In the present study, NMDAR protein expression in various brain regions was examined from separate groups of F639A and wild-type mice following voluntary ethanol consumption or repeated non-contingent exposures to ethanol. We also examined whether locomotor sensitization that develops in some strains during repeated exposures to ethanol, was altered by the F639A mutation and whether changes in NMDAR subunit expression were correlated with these effects.

## Methods

### Mice

Adult (>60 days old) male wild-type and F639A mice on a mixed C57/S129 background were used in all of the studies. Mice were generated by Het × Het breeding as described previously (den Hartog et al., 2013) and were of the N2 generation. Mice were group housed unless otherwise noted and had free access to chow and water. This study was carried out in accordance with the recommendations of the NIH's Guidelines for the Care and Use of Laboratory Animals (8th edition). The protocol was approved by the MUSC Institutional Animal Care and Use Committee.

### Ethanol treatments

Protein expression was examined in mice that had undergone one of three different exposure protocols. In the first study, tissue was collected from wild-type and F639A mice following long-term (85 days) voluntary drinking using the intermittent (every other day) access model. These mice were singly housed and during the first half of the drinking study, had access every other day to a bottle containing 20% ethanol plus 0.2% saccharin or a bottle of water. During the second half of the study, the ethanol concentration was increased to 40% (with 0.2% saccharin). On non-drinking days, mice had access to two bottles of water. The drinking data for these animals was previously reported by den Hartog et al. (2013) and brain tissue was collected 24 h after the last drinking session. A separate group of wild-type and F639A mice received 8 injections of saline or 18% ethanol (3 g/kg; i.p.) with injections occurring every other day. Animals were sacrificed 24 h following the last injection for tissue collection. A third group of animals were treated with twice daily injections of saline or 18% ethanol (2 g/kg or 3.5 g/kg; i.p.) for 10 days. Mice were sacrificed 24 h after the last injection and tissue was collected for western blot analysis.

### Locomotor response to repeated ethanol treatment

Mice were habituated to handling in the testing room for 3 days before the start of the experiment and were habituated to the room for at least 30 min prior to each locomotor test or injection. Locomotor activity was always measured immediately following an injection. Baseline locomotor activity was measured on two separate days following treatment with saline. Mice were then split into either saline or ethanol (1.5 g/kg) treatment groups and tested for locomotor activity following injection on days 1, 3, 5, 7, 9, 17, 25, and 35. On the intervening days, mice were injected according to their treatment group (ethanol or saline) in the same testing room but locomotor activity was not measured. Total distance traveled was recorded using Any-Maze (ANYmaze, Stoelting Co., Wood Dale, IL) tracking software.

### Western blotting

N-Methyl-D-Aspartate receptor (NMDAR) subunit expression in mice was analyzed by western blotting as previously described (Pava et al., 2012). Briefly, animals were rapidly euthanized by decapitation, and brains were immediately immersed for 1–2 min in ice-cold dissection buffer containing (in mM): sucrose (200), KCl (1.9), MgCl_2_ (6), CaCl_2_ (0.5), glucose (10), ascorbic (0.4) acid, HEPES (25), pH 7.3 with KOH. Brains were sectioned into 1–2 mm thick coronal slices using an adult mouse brain matrix (ASI Instruments, Warren, MI) and brain punches were isolated from 6 brain regions (orbitofrontal cortex, OFC; medial prefrontal cortex, mPFC; dorsal striatum, DS; nucleus accumbens, NAcc; hippocampus, HC; and basolateral amygdala, BLA) from each mouse. Punches were hand-homogenized in ice-cold homogenization buffer [50 mM Tris-HCl, 50 mM NaCl, 10 mM EGTA, 5 mM EDTA; 2 mM Na+ pyrophosphate, 1 mM activated Na+ orthovanadate, 1 mM Na+ fluoride, pH 7.5, containing Halt Protease Inhibitor Cocktail (Thermo Scientific, Waltham, MA)], sonicated by probe and centrifuged at 23,000 × g for 30 min at 4°C. The supernatant was removed from each sample and the remaining pellet was solubilized in 2% sodium dodecyl sulfate (SDS) using the sonic probe. Protein concentration of the sample was determined using bicinchoninic acid assay (Pierce Biotechnology, Inc., Rockford, IL). Antibodies used in these studies were GluN1 (BD Pharmingen, Franklin Lakes, NJ), GluN2A (Millipore, Billerica, MA) and GluN2B (NeuroMab, Antibodies Inc., & UC Davis, Davis, CA). Antibody protein bands were detected by enhanced chemiluminescence using a ChemiDoc MP Imaging system (Bio-Rad Laboratories, Hercules, CA, USA). The band corresponding to each appropriate subunit was quantified by mean optical density using computer-assisted densitometry with ImageJ v1.41 (National Institutes of Health, USA). Due to reports of quantitation errors associated with common loading controls, such as β-actin (Dittmer and Dittmer, 2006; Aldridge et al., 2008) and the potential for ethanol-induced changes in some of these proteins (Alexander-Kaufman et al., 2006; Ou et al., 2009, 2011), loading controls were not used. Instead, all blots contained replicates of each study's experimental groups and following background subtraction of image intensity, data was calculated as a percent of wild-type or saline controls run simultaneously on each blot. During analysis, the experimenter was blinded to the treatment condition of each lane. In some experiments, total protein was stained after transfer using Swift Membrane Stain according to manufacturer's protocol (G-Biosciences, St. Louis, MO). As shown in Supplemental Figure 1, data from the background-subtracted blots were nearly identical to that obtained using the total protein stain.

### Statistics

Data from western blot experiments and the locomotor sensitization study were analyzed with SPSS (v.23, IBM, Armonk, NY) using a linear mixed model with significance indicated when *p* < 0.05. Pairwise comparisons were Bonferroni corrected where applicable.

## Results

### Protein expression following consumption of ethanol

**Figure 1:**
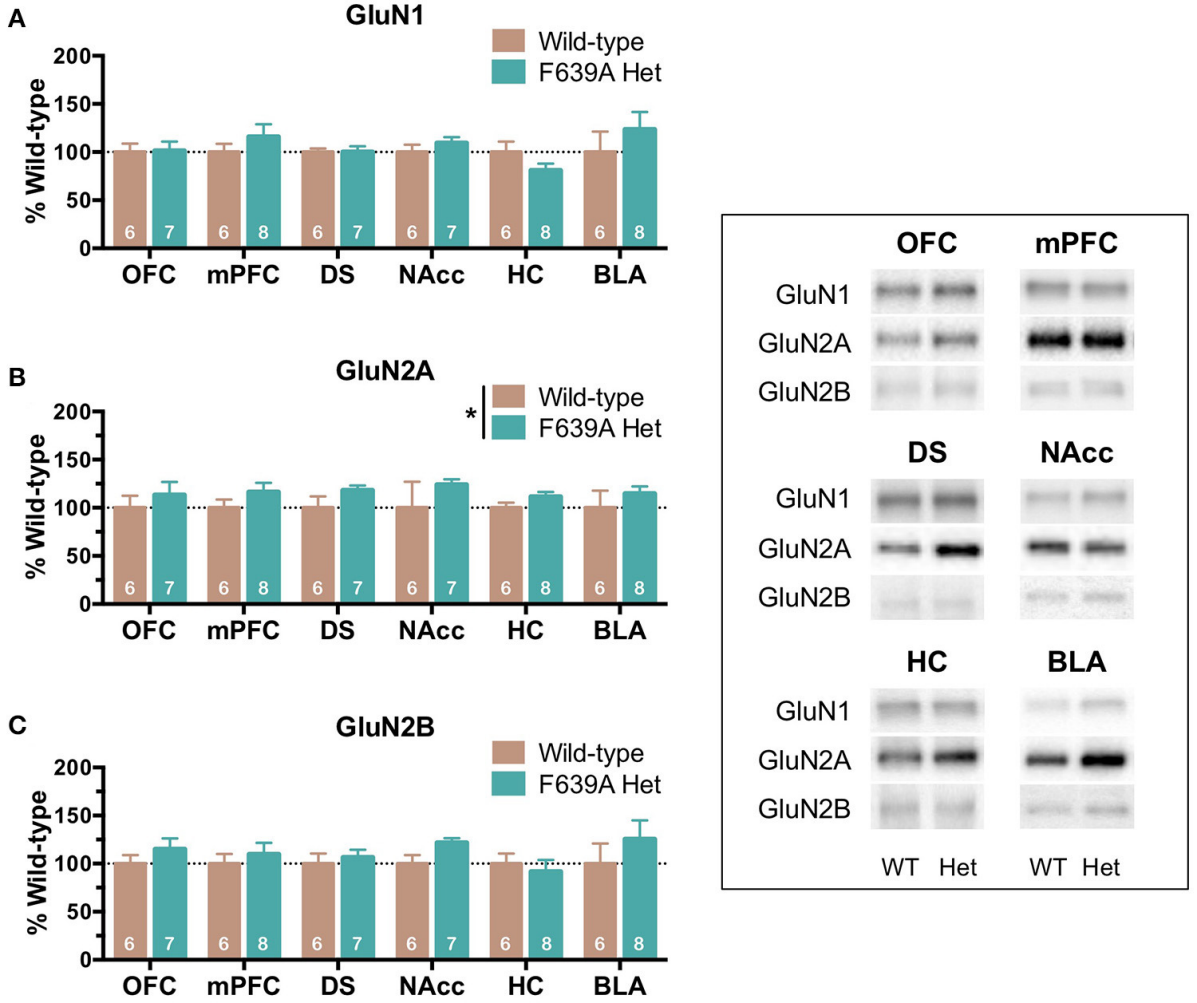
**Effects of long-term ethanol consumption on NMDAR subunit expression in wild-type and F639A mice**. Tissue was collected from mice used in a drinking study reported in den Hartog et al. (2013). In that study, wild-type and F639A C57/S129 male mice were given 24 h access to increasing concentrations of ethanol (3–15%; plus 0.2% saccharine) followed by 5 weeks of 20% ethanol (plus 0.2% saccharin) and 5 weeks of 40% ethanol (plus 0.2% saccharin). Twenty-four hours after the last drinking session, tissue punches from orbitofrontal cortex (OFC), medial prefrontal cortex (mPFC), dorsal striatum (DS), nucleus accumbens (Nacc), hippocampus (HC), and basolateral amygdala (BLA) were collected and analyzed for GluN1 **(A)**, GluN2A **(B)**, and GluN2B **(C)** subunit expression. Mean (± SEM; N) optical density values for protein bands are shown as percent of wild-type controls run the in same blot. Symbol: significant effect of genotype on protein expression of GluN2A (^*^p < 0.05). Inset shows representative example of western blot showing NMDA subunit expression in wild-type (WT) and F639A (Het) mice.

Brain tissue from wild-type and F639A Het mice that underwent long-term drinking was collected 24 h after the last drinking session and analyzed for differences in NMDAR protein expression. As previously reported (den Hartog et al., 2013), F639A Het mice consumed more ethanol than wild-type mice when offered ethanol in 0.2% saccharin. Over the 85 day period, total ethanol consumption was (in g/kg, mean ± SEM); 330.1 ± 51.8; and 405.9 ± 36.7; respectively for wild-type and F639A mice. As shown in Figure 1, analysis of brain tissue from these mice collected 24 h following their last drinking session revealed a main effect of genotype on GluN2A expression [*F*_(1, 287)_ = 5.53, *p* = 0.019] with F639A mice showing an overall increase over WT mice and a trend for an increase in GluN2B expression [*F*_(1, 287)_ = 2.93, *p* = 0.088] for F639A mice. No significant differences between WT and F639A mice were noted for expression of GluN1 [*F*_(1, 287)_ = 0.96, *p* = 0.329].

### Protein expression following repeated ethanol injections

**Figure 2:**
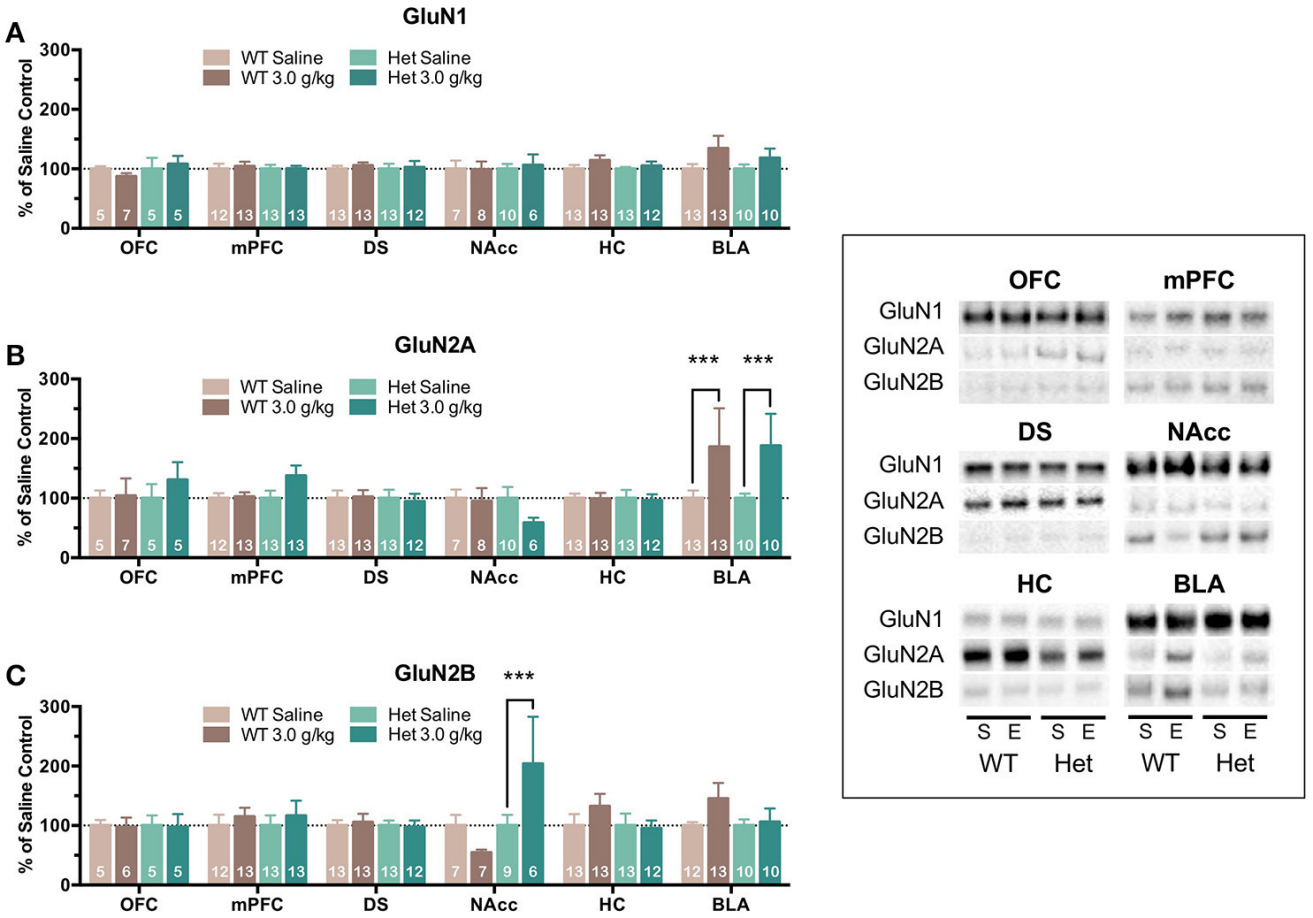
**Effects of repeated ethanol treatment on NMDAR subunit expression in wild-type and F639A mice**. Western blot analysis of GluN1 **(A)**, GluN2A **(B)**, GluN2B **(C)** subunit expression from wild-type and F639A mice treated with 8 injections of saline (S) or ethanol (E; 3.0 g/kg) administered every other day. Tissue was collected 24 h following the last injection from orbitofrontal cortex (OFC), medial prefrontal cortex (mPFC), dorsal striatum (DS), nucleus accumbens (NAcc), hippocampus (HC), and basolateral amygdala (BLA). Mean (±SEM) optical density values for protein bands are shown as percent of corresponding saline controls run the in same blot. Symbols: value significantly different from corresponding saline injected mice (^***^p < 0.001). Inset shows representative example of western blot showing NMDA subunit expression in saline (S) and ethanol (E) treated wild-type (WT) and F639A (Het) mice.

**Figure 3:**
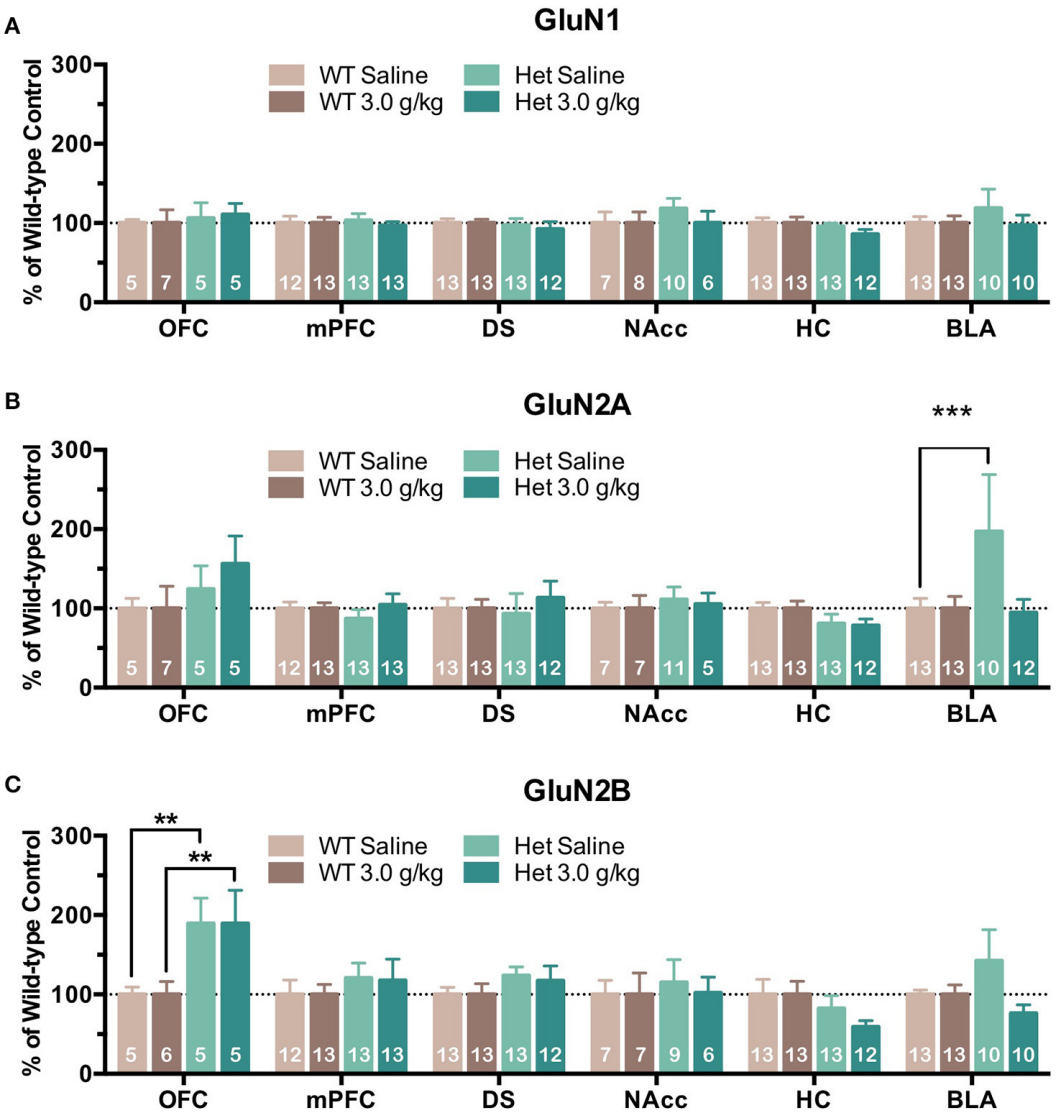
**Effects of genotype on NMDAR subunit expression following repeated non-contingent ethanol treatment**. Western blot analysis of GluN1 **(A)**, GluN2A **(B)**, GluN2B **(C)** subunit expression from wild-type and F639A mice treated with 8 injections of saline or ethanol (3.0 g/kg) administered every other day. Tissue was collected 24 h following the last injection from orbitofrontal cortex (OFC), medial prefrontal cortex (mPFC), dorsal striatum (DS), nucleus accumbens (NAcc), hippocampus (HC), and basolateral amygdala (BLA). Mean (±SEM) optical density values for protein bands are shown as percent of the corresponding wild-type controls run the in same blot. Symbol: value significantly different from control (^**^p < 0.01, ^***^p < 0.001).

Although free-choice drinking in mice is a more human-like model of ethanol drinking, it does not control for the amount of ethanol administered and as reported above, F639A mice showed elevated volitional consumption of ethanol. To eliminate this variable, wild-type and F639A mice were exposed to repeated injections of ethanol or saline and protein expression of NMDAR subunits was measured. In the first treatment protocol, mice received an injection of ethanol (3 g/kg, i.p.) or saline every other day for a total of 8 injections. Mean optical density of each band corresponding to the GluN1, GluN2A, GluN2B subunit for each brain region is shown as the percent of saline or percent of wild-type controls that were run simultaneously in the same blot. For data expressed as a percent of saline (Figure 2), there were no significant effects of ethanol treatment on GluN1 expression for either genotype. In contrast, both ethanol-treated WT and F639A mice showed a significant increase in expression of GluN2A in the BLA [WT *F*_(1, 675)_ = 13.0, *p* = 0.0003; F639A *F*_(1, 675)_ = 10.94, *p* = 0.001] as compared to their saline treated counterparts. Expression of GluN2B was significantly increased following ethanol treatment in the NAcc of F639A mice [*F*_(1, 675)_ = 13.17, *p* = 0.0003] while WT mice showed a non-significant trend for a decrease [*F*_(1, 675)_ = 1.82, *p* = 0.178]. WT mice also showed a trend for increased GluN2B expression in the BLA but this was also not statistically significant [*F*_(1, 675)_ = 3.41, *p* = 0.065]. Data normalized to the corresponding WT controls (Figure 3) was used to test for genotype-dependent effects on expression. Statistical analysis of this data revealed no significant effect of F639A on expression of GluN1 for either saline or ethanol treatment conditions. As compared to WT mice, saline-treated F639A mice had higher GluN2A expression in the BLA [*F*_(1, 673)_ = 17.80, *p* = 0.00003] but in ethanol-treated mice there was no difference between the two genotypes. In addition, there was a near-significant trend [*F*_(1, 673)_ = 3.09, *p* = 0.08] for an increase in GluN2A expression in the OFC of ethanol-treated F639A mice as compared to ethanol-treated WT controls. For GluN2B, saline-treated F639A mice showed a significant increase over WT mice in the OFC [*F*_(1, 673)_ = 6.68, *p* = 0.01] and a trend for increased expression in the BLA [*F*_(1, 673)_ = 2.89, *p* = 0.09]. Following ethanol treatment, F639A mice showed a significant elevation in GluN2B expression in the OFC [*F*_(1, 673)_ = 7.30, *p* = 0.007] while a trend for a decrease was observed in the hippocampus [*F*_(1, 673)_ = 3.34, *p* = 0.07].

**Figure 4:**
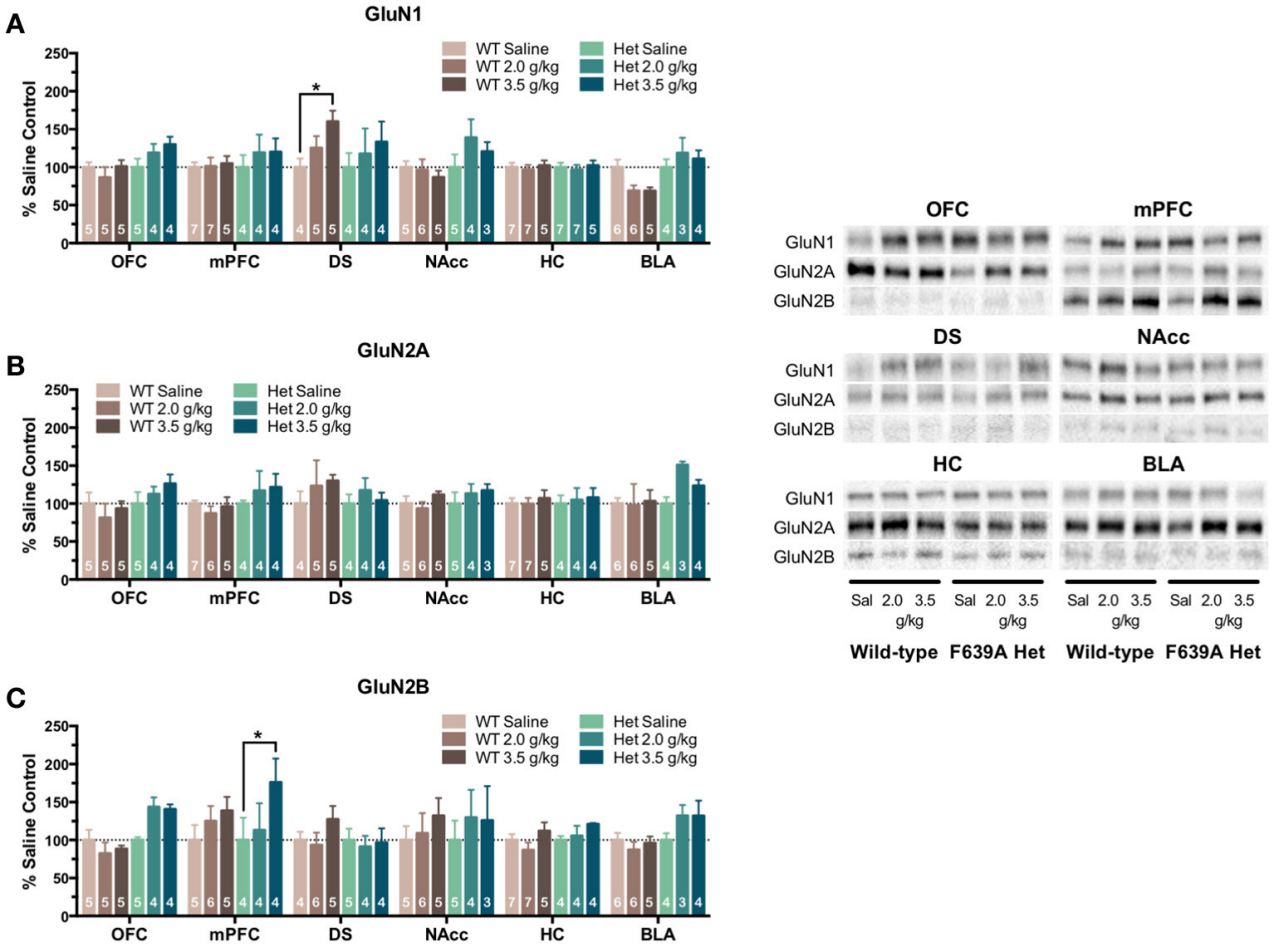
**Effects of repeated ethanol treatment on NMDAR subunit expression in wild-type and F639A mice**. Western blot analysis for GluN1 **(A)**, GluN2A **(B)**, GluN2B **(C)** subunit expression from saline or ethanol-treated wild-type and F639A mice. Mice were treated with twice daily injections of ethanol (2.0 or 3.5 g/kg) or saline for 10 consecutive days. Tissue was collected 24 h following the last injection from orbitofrontal cortex (OFC), medial prefrontal cortex (mPFC), dorsal striatum (DS), nucleus accumbens (NAcc), hippocampus (HC), and basolateral amygdala (BLA). Mean optical density values for protein bands are shown as percent of saline controls run the in same blot. Symbol: value significantly different from control (^*^p < 0.05). Inset shows representative example of western blot showing NMDA subunit expression in saline (S) and ethanol (E) treated wild-type (WT) and F639A (Het) mice.

**Figure 5:**
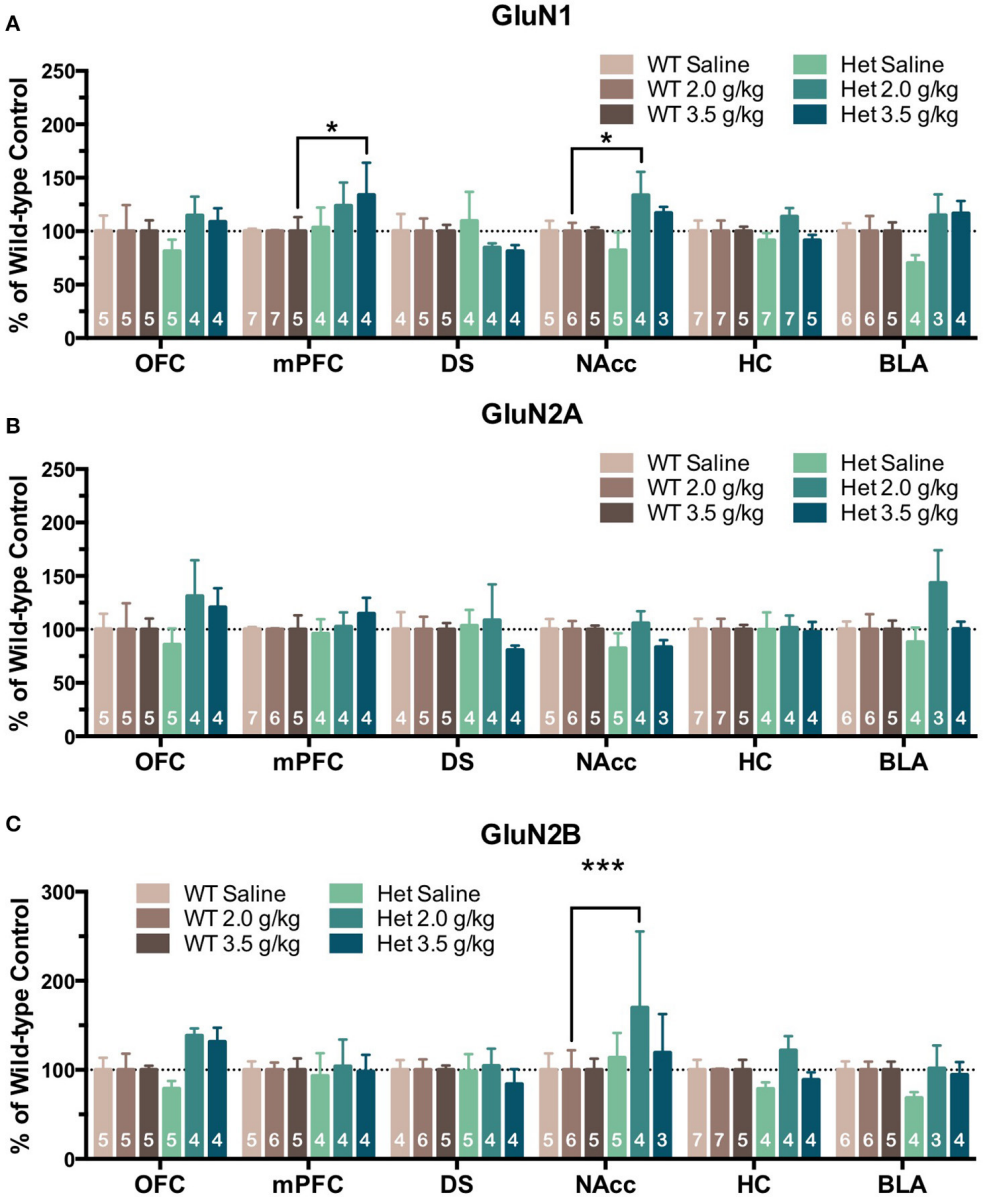
**Effects of genotype on NMDAR subunit expression following repeated non-contingent ethanol treatment**. Western blot analysis for GluN1 **(A)**, GluN2A **(B)**, GluN2B **(C)** subunit expression from saline or ethanol-treated wild-type and F639A mice. Mice were treated with twice daily injections of ethanol (2.0 or 3.0 g/kg) or saline for 10 consecutive days. Tissue was collected 24 h following the last injection from orbitofrontal cortex (OFC), medial prefrontal cortex (mPFC), dorsal striatum (DS), nucleus accumbens (NAcc), hippocampus (HC), and basolateral amygdala (BLA). Mean optical density values for protein bands are shown as percent of wild-type controls run the in same blot. Symbol: value significantly different from control (^*^p < 0.05; ^***^p < 0.001).

In the second treatment paradigm, separate groups of mice were injected with either saline or ethanol (2.0 or 3.5 g/kg) twice a day for 10 days. Mean optical density of each band corresponding to the GluN1, GluN2A and GluN2B subunit is shown as the percent of saline (Figure 4) or wild-type controls (Figure 5) run simultaneously in the same blot. A significant effect of ethanol treatment was observed for GluN1 expression in the DS [*F*_(2, 445)_ = 3.89, *p* = 0.021] of wild-type mice and pairwise comparisons revealed a significant increase in GluN1 expression by the 3.5 g/kg dose as compared to saline (*p* = 0.018; Figure 4A). There was also a trend for ethanol to decrease GluN1 expression in the BLA of wild-type mice [*F*_(2, 445)_ = 2.46, *p* = 0.087] but pairwise comparisons did not reach statistical significance. No significant change in GluN1 expression was found in ethanol-treated F639A mice as compared to saline controls. Similarly, there were no significant changes in expression of GluN2A in any region of ethanol-treated wild-type or F639A mice (Figure 4B). Ethanol treatment also did not significantly affect GluN2B expression in any region of wild-type mice (Figure 4C) while in F639A mice, GluN2B levels were significantly elevated in the mPFC [*F*_(2, 445)_ = 3.62, *p* = 0.028; pairwise comparison control vs. 3.5 g/kg *p* = 0.023] and showed a trend for an increase in the OFC [*F*_(2, 445)_ = 2.59, *p* = 0.077]. When data from these studies were expressed as percent of the corresponding wild-type control to examine genotype-dependent effects, region-specific changes were noted for the different subunits. As compared to saline-treated wild-type mice, F639A mice showed a trend for decreased expression of GluN1 in the BLA [*F*_(1, 416)_ = 3.14, *p* = 0.077] while GluN1 was higher in the mPFC of F639A mice treated with 3.5 g/kg ethanol [*F*_(1, 416)_ = 4.56, *p* = 0.033; Figure 5A]. No significant effects were noted for GluN2A expression although there was a near-significant trend for increased expression in the BLA of F639A mice treated with 2 g/kg ethanol [*F*_(1, 416)_ = 3.77, *p* = 0.053; Figure 5B]. For GluN2B, there was a significant increase in expression in the NAcc of F639A mice treated with 2 g/kg ethanol [*F*_(1, 416)_ = 59.96, *p* < 0.0001] with a trend toward an increase in the OFC [*F*_(1, 416)_ = 3.26, *p* = 0.072] from the same animals (Figure 5C).

### Locomotor activity and protein expression following repeated ethanol injections

**Figure 6:**
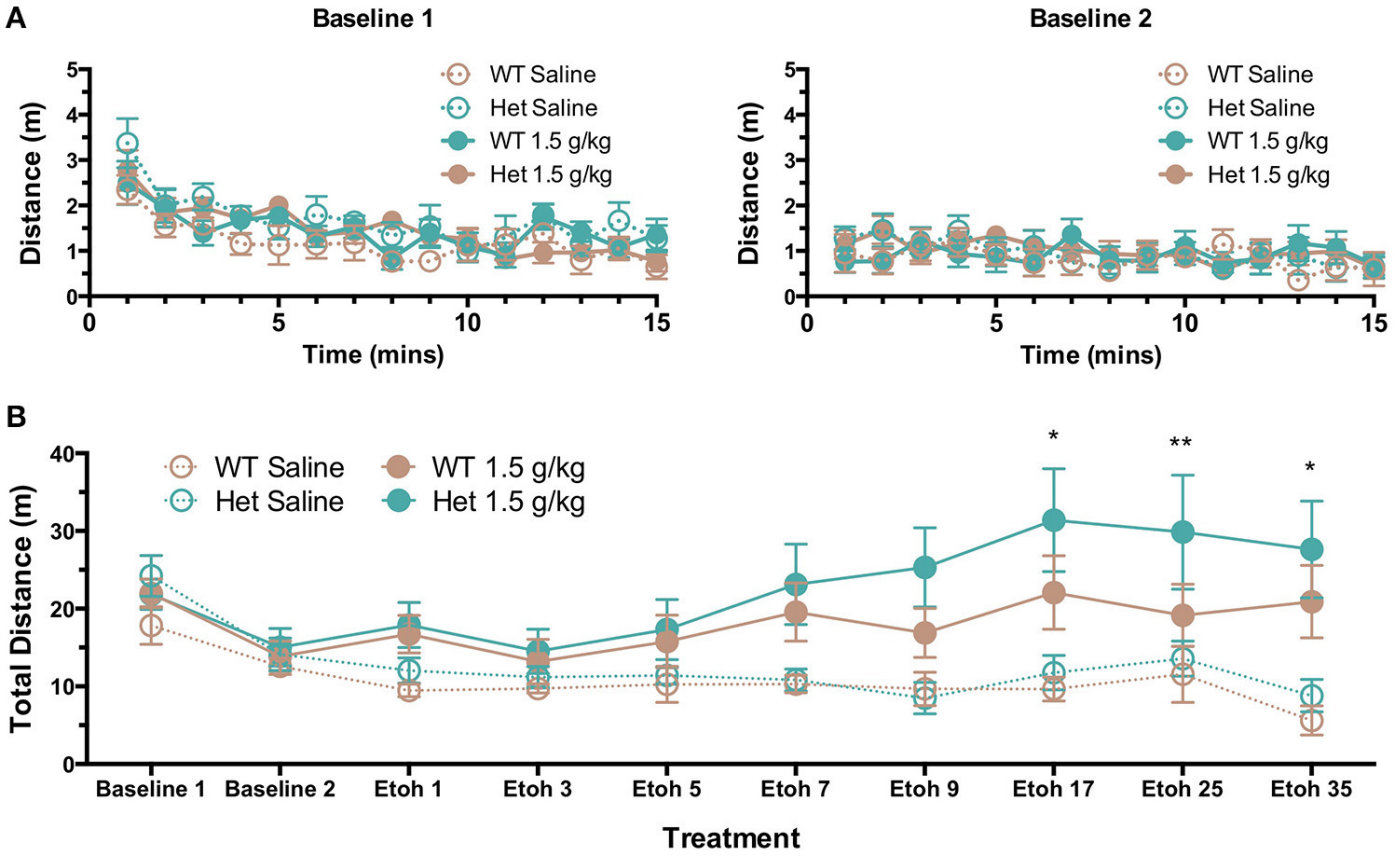
**Effects of repeated ethanol injections on locomotor activity of wild-type and F639A mice. (A)** Baseline locomotor activity in mice following saline injections on two consecutive test days (top panels). **(B)** Locomotor activity in mice receiving daily injections of saline or ethanol (1.5 g/kg; i.p.). Mice received injections every day and activity was measured on days 1, 3, 5, 7, 9, 17, 25, and 35. Total distance traveled (m) was measured for 15 min immediately following injection. Symbols: value different from test day 3 (^*^p < 0.05; ^**^p < 0.01).

Given the previously reported differences in locomotor response of wild-type and F639A mice to acute ethanol (den Hartog et al., 2013), we sought to determine if locomotor sensitization that often develops following repeated injections would differ between WT and F639A mice. Baseline locomotor activity following an initial injection of saline was not different between F639A Het and wild-type mice and both groups showed a novelty-induced increase in locomotor activity that was absent on the second day of baseline testing (Figure 6A). Wild-type and F639A mice treated with repeated injections of saline showed no changes in locomotor activity throughout the study (test days 1–35; Figure 6B). However, following repeated treatment with ethanol, F639A mice but not WT showed a significant increase in locomotor activity as a function of test day [*F*_(7, 150.24)_ = 4.77, *p* = 0.00007]. Pairwise comparisons revealed significant differences in locomotor activity for F639A mice at test days 17 (*p* = 0.017), 25 (*p* = 0.007), and 35 (*p* = 0.05) as compared to test day 3.

**Figure 7:**
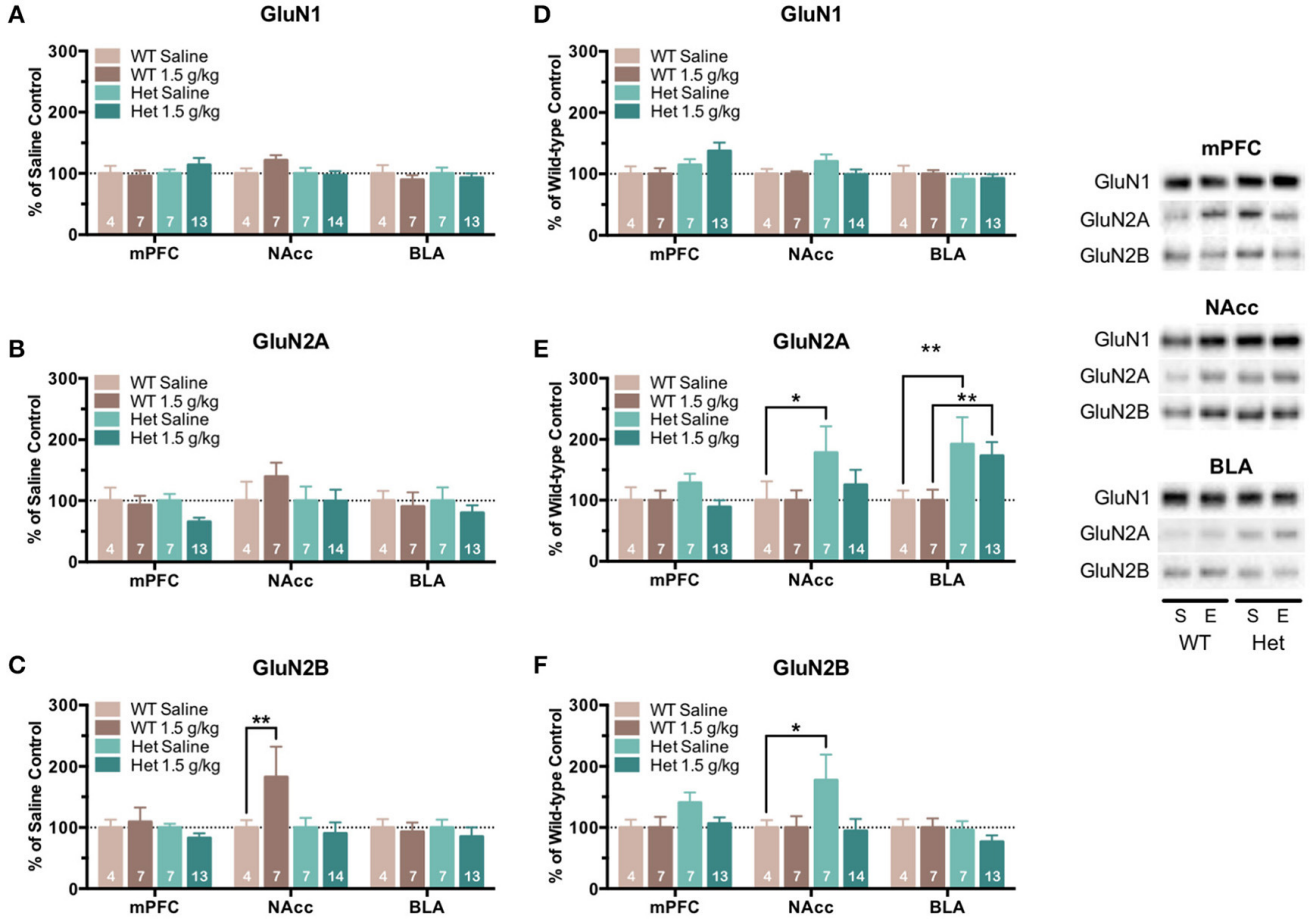
**NMDAR subunit expression in mice tested for locomotor sensitization to ethanol**. One day following the last ethanol injection, tissue was isolated from the medial prefrontal cortex (mPFC), nucleus accumbens (NAcc), and basolateral amygdala (BLA) of wild-type and F639A mice and was analyzed for GluN1 **(A)**, GluN2A **(B)**, and GluN2B **(C)** subunit expression. Mean (±SEM) optical density values for protein bands are shown as percent of corresponding saline controls **(A–C)** or wild-type controls **(D–F)** run the in same blot. Symbol: value significantly different from corresponding control (^*^p < 0.05; ^**^p < 0.01). Inset shows representative example of western blot showing NMDA subunit expression in saline (S) and ethanol (E) treated wild-type (WT) and F639A (Het) mice.

Following locomotor sensitization testing, animals were sacrificed and brain tissue was collected from select brain regions (mPFC, NAcc, and BLA) and analyzed for NMDAR subunit expression. Mean optical density of each band corresponding to the GluN1, GluN2A, GluN2B subunit is shown as the percent of saline (Figures 7A–C) or percent of wild-type controls (Figures 7D–F) run simultaneously in the same blot. As compared to saline-treated controls, wild-type mice treated with ethanol showed a significant increase in the expression of GluN2B in the NAcc [*F*_(1, 243)_ = 8.08, *p* = 0.005; Figure 7C] with no changes in GluN1 (Figure 7A) or GluN2A (Figure 7B) expression in any region. As compared to saline controls, F639A mice showed no change in expression of any GluN subunit (Figures 7A–C) following ethanol treatment although levels of GluN2A in the mPFC showed a trend toward a decrease. When expressed as a percent of their corresponding wild-type control, F639A mice showed significant changes in expression of GluN2A and GluN2B that were region-dependent. While no genotype-dependent changes in GluN expression were observed in the mPFC, levels of GluN2A [*F*_(1, 243)_ = 5.20, *p* = 0.023; Figure 7E] and GluN2B [*F*_(1, 243)_ = 5.12, *p* = 0.025; Figure 7F] in the NAcc were higher in saline-treated F639A animals as compared to wild-type mice but not those treated with ethanol. In the BLA, F639A mice had higher expression of GluN2A following both saline [*F*_(1, 243)_ = 7.29, *p* = 0.007; Figure 7E] and ethanol [*F*_(1, 243)_ = 8.13, *p* = 0.005; Figure 7E] treatment with no changes in levels of GluN1 or GluN2B.

## Discussion

### The F639A mutation does not prevent ethanol-induced changes in NMDAR expression

In our previous studies, we reported that the GluN1(F639A) mutation significantly reduces ethanol inhibition of recombinant NMDARs expressed in heterologous cells (Ronald et al., 2001; Smothers and Woodward, 2006). NMDA EPSCs from mice carrying the F639A mutation also have reduced ethanol sensitivity and these animals show differences in various behavioral responses to ethanol including changes in ethanol consumption (den Hartog et al., 2013). Based on these findings, we hypothesized that changes in NMDAR expression that often follow repeated exposures to ethanol would be blunted in F639A mice due to the reduced ethanol inhibition of these receptors. The results of the present study reveal an unexpected degree of complexity with respect to ethanol-induced changes in NMDAR expression and show an interaction between genotype, brain region and ethanol exposure protocol. Overall, the results of this study also suggest that the intrinsic ethanol sensitivity of NMDARs is not the sole factor that drives changes in the expression of NMDA subunits following chronic exposure to ethanol.

### Changes in NMDAR expression are selective and can occur in an ethanol-independent manner

Analysis of protein expression from mice that underwent long-term drinking shows that F639A Het had higher levels of GluN2A expression across all brain regions examined (OFC, mPFC, DS, NAcc, HC, and BLA) as compared to their wild-type counterparts. In these same animals, there was also a trend for higher expression of GluN2B subunits although not all regions showed these changes. As reported by den Hartog et al. (2013), naïve F639A mice that have not been exposed to handling or ethanol express similar amounts of NMDAR subunits across various brain regions (mPFC, DS, HC, and BLA, NAcc) as their wild-type littermates with the exception of a small but significant decrease in GluN2A in the mPFC. The increased expression of NMDAR subunits in F639A mice following long-term access to ethanol noted in the present study may be one factor that contributes to the maintenance of elevated drinking in these animals. However, an important caveat with these studies is that the F639A mice drank slightly more ethanol (~23%) than wild-type animals and this increase in consumption may have driven the change in receptor expression rather than factors related to the genotype. To address this issue, additional studies using non-contingent delivery were conducted in order to match the amount of ethanol experienced by the two strains.

The results from the forced ethanol injection protocols suggest that NMDA subunits show alterations in expression that are affected both by the genotype and the exposure protocol used. In the first study that utilized 8 injections of ethanol (3.0 g/kg, ip) every other day, the BLA and the NAcc were the most responsive to changes in expression and of the three subunits examined, GluN2A and GluN2B subunits appeared to be more affected by the F639A mutation than GluN1. In the second study that used twice-daily injections over a 10-day period, changes were found across all three subunits and were observed in the DS, mPFC and NAc. GluN2B seemed most affected by ethanol treatment in both groups, and GluN1 expression appeared to differ more between F639A and wild-type mice. When results of the two studies are examined together, the mPFC, the BLA, and the NAcc appear to be the regions most likely to show genotype—and treatment—induced differences. This finding is consistent with various reports in the literature suggesting that these regions are involved in various aspects of drug and alcohol addiction (Steketee and Kalivas, 2011) and neurons within these areas often show alterations in function following chronic exposure to ethanol (Roberto et al., 2004; Kroener et al., 2012; Abrahao et al., 2013). Interestingly and as discussed above, our previous report showed that expression of NMDAR subunits is similar between naïve F639A and wild-type mice (den Hartog et al., 2013), while in the present study, there were several genotype-dependent differences in expression in saline-treated animals. Although not yet directly tested, these findings suggest that in addition to any differences in their response to ethanol, F639A and wild-type mice may differ in their sensitivity to the stress associated with repeated episodes of handling and injection. This could reflect subtle alterations in receptor function that are associated with the F639A mutation, such as the heightened sensitivity to the co-agonist glycine observed in recombinant receptors (Ronald et al., 2001; Smothers and Woodward, 2006) and faster responses to exogenous agonists observed in brain slice studies (den Hartog et al., 2013). Behaviorally, F639A mice show similar responses following an injection of saline in a test of anxiety (zero-maze den Hartog et al., 2013) although this involved only a single injection rather than the extensive series of injections mice received in the present study. While additional studies are needed to fully clarify this issue, these data suggest that mutations designed to selectively alter ethanol sensitivity of selected proteins may also produce effects on behaviors and function that can be observed in the absence of ethanol.

### Ethanol-resistant NMDARs may favor locomotor sensitization to ethanol

In the locomotor sensitization study, F639A but not wild-type mice showed a progressive increase in locomotor activity following repeated injections with ethanol. The lack of ethanol sensitization in wild-type mice in the present study likely reflects their C57Bl/6 background as these mice do not typically show locomotor sensitization following repeated exposures to ethanol (Cunningham et al., 1992; Phillips et al., 1994). Interestingly, it has been shown previously that pre-treatment of mice with the NMDA antagonist MK-801 blocks the development of locomotor sensitization to ethanol (Broadbent and Weitemier, 1999; Camarini et al., 2000). These findings indicate that unlike changes in NMDAR subunit expression that were not clearly genotype-dependent, the degree of ethanol inhibition of NMDARs is a critical factor that influences the development of sensitization. Based on the MK-801 data and reports from the literature, C57Bl/6 mice expressing ethanol-resistant NMDARs would be more likely to show locomotor sensitization, consistent with what was observed for the F639A mice used in the present study. The precise locus that underlies ethanol-induced locomotor sensitization is not completely known but sensitization to psychostimulants, such as cocaine and amphetamine is known to involve mesocorticolimbic circuitry (e.g., VTA, NAc, mPFC) as well as other areas, such as the BLA and paraventricular nucleus (PVN) that interact with these regions (reviewed by Steketee and Kalivas, 2011). Changes in NMDAR expression following repeated exposures to cocaine have also been reported, although like for ethanol, these effects depend on the region examined, the exposure protocol and the time following withdrawal (reviewed by Ortinski, 2014). In general, areas, such as the NAc, VTA and BLA seem especially prone to alterations in NMDAR expression following cocaine exposure whereas areas, such as the mPFC appear to be less sensitive (Ortinski, 2014). These findings are generally consistent with those of the present study where alterations in NMDAR expression following the ethanol sensitization protocol were observed in the NAc and BLA but not the mPFC. Interestingly, significant changes in NMDAR subunit expression following the repeated ethanol injections occurred only in wild-type mice that did not show locomotor sensitization. A similar finding was reported for changes in NMDAR subunit mRNA expression in which mice functionally characterized as low-sensitized showed increases in GluN subunits in various regions including the NAc while levels in sensitized mice were not different from controls (Nona et al., 2014). These authors also showed that changes in mRNA expression were only observed in mice sacrificed 1 day following the last ethanol injection as no differences in GluN mRNA were found in mice examined 14 days following the last injection.

In a similar study, Abrahao et al. (Abrahao et al., 2013) examined ethanol drinking and electrophysiological properties of NAc neurons in ethanol-sensitized and non-sensitized mice and compared these to changes in NMDAR expression in the NAc. They reported that ethanol-sensitized mice drank more ethanol than non-sensitized mice and had an elevated AMPA/NMDA ratio in NAc neurons that appeared to be due to reduced NMDA signaling. This was accompanied by a blunted NMDA-dependent long-term depression (LTD) and subunit-dependent changes in NMDAR expression in the NAc with decreases in GluN1 and GluN2A and an increase in GluN2B (Abrahao et al., 2013). The changes in NMDAR expression are somewhat different from those reported in the present study and that of Nona et al. (2014) and may reflect differences in mouse strain (Swiss-Webster vs. C57/S129 vs. DBA2), number of ethanol injections (21 vs. 35 vs. 5–6) and timing of tissue collection (2 weeks after last injection; 24 h after last injection; 24 h-2 weeks) between the three studies. Together, while the results of the previous studies confirm that changes in NMDAR expression following ethanol exposure are dynamic and complex, findings from the present study suggest that while reducing the intrinsic ethanol sensitivity of NMDARs does not eliminate these changes, differences in acute sensitivity to ethanol may be one mechanism that contributes to the development of locomotor sensitization and escalations in drinking. Whether findings from these pre-clinical studies can be extended to humans in order to better predict who is at risk for developing an AUD remains to be determined as polymorphisms at ethanol-sensitive sites in human NMDARs have not yet been observed. However, the ethanol sensitivity of NMDARs also varies as a function of post-translational modifications (Anders et al., 1999; Xu and Woodward, 2006; Xu et al., 2011) and the presence of different NMDAR splice variants (Jin and Woodward, 2006) suggesting that non-genomic mechanisms may also contribute to individual differences in susceptibility.

In summary, the results of the present study suggest that changes in NMDAR subunit expression and behavior following chronic exposure to ethanol is likely not a simple homeostatic response to receptor inhibition.

## Author contributions

GH generated the F639A mice; Cd and JW designed the experiments; Cd, MG, BE, DL, and PM performed the experiments; Cd, MG, BE, PM, and JW analyzed the data; Cd and JW wrote the paper.

## Funding

This work was supported by grants R37AA009986 (JW), U01AA20930 (PM), R37AA10422 (GH) and an MUSC Neuroscience Institute International Fellowship (Cd).

### Conflict of interest statement

The authors declare that the research was conducted in the absence of any commercial or financial relationships that could be construed as a potential conflict of interest.
