# Supplementary material for: Effects of Repeated Ethanol Exposures on NMDA Receptor Expression and Locomotor Sensitization in Mice Expressing Ethanol Resistant NMDA Receptors
Source: Front Neurosci. 2017 Feb 21;11:84. doi: 10.3389/fnins.2017.00084 (PMC5318453; doi:10.3389/fnins.2017.00084)

Supplemental Figure 1. Comparison of western blot quantitation using either a total protein stain (Swift Stain) or background subtraction. Figures show expression of NMDA subunits from the locomotor sensitization study calculated as either percent of saline control (top panel) or percent of wild-type control (bottom panel). In each figure, blots were quantitated using either a total protein stain (left side) or background subtraction (right side). Data shown in the background subtraction figures (right panel in each figure) are the same as in Figure 7 of the manuscript.


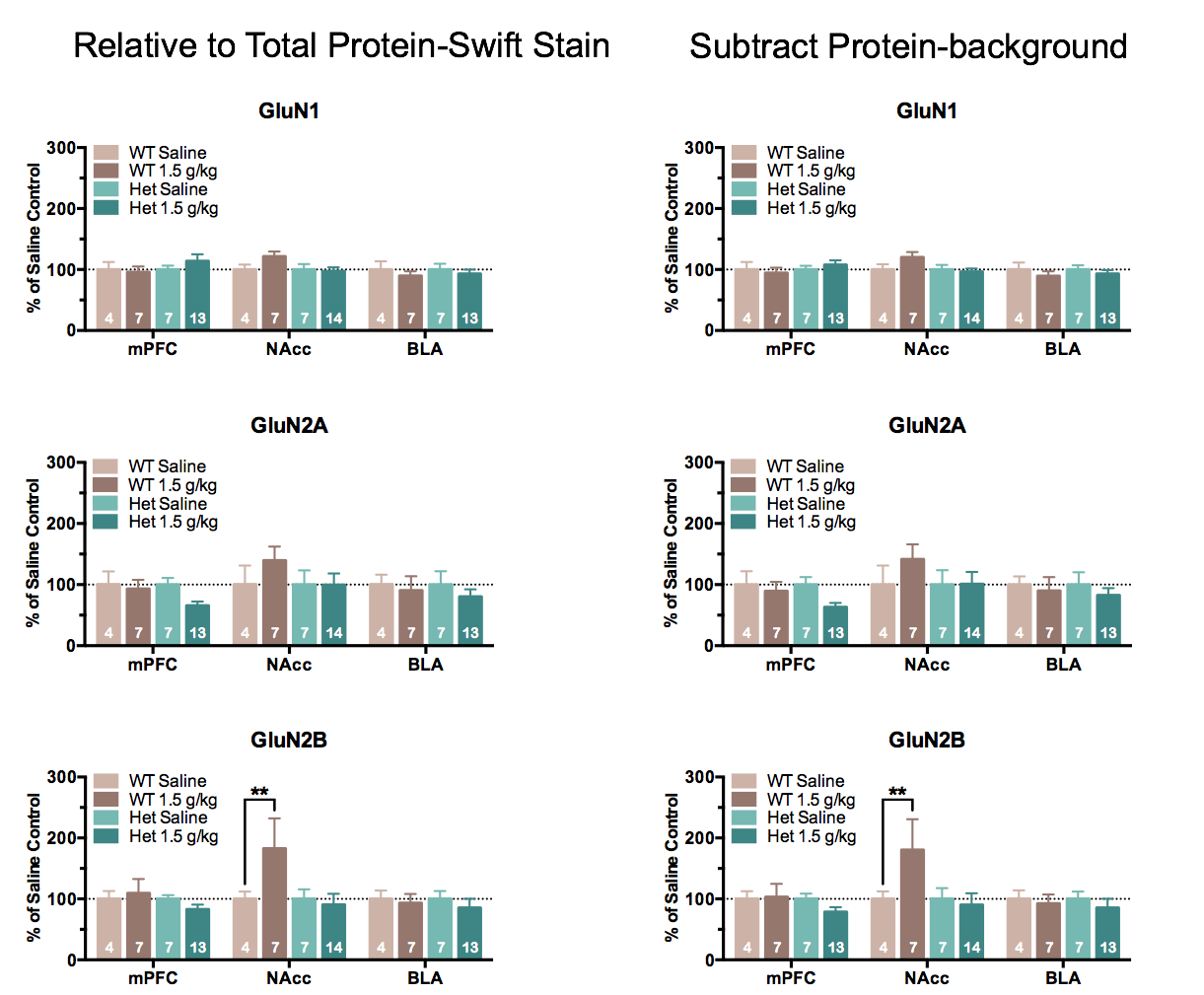


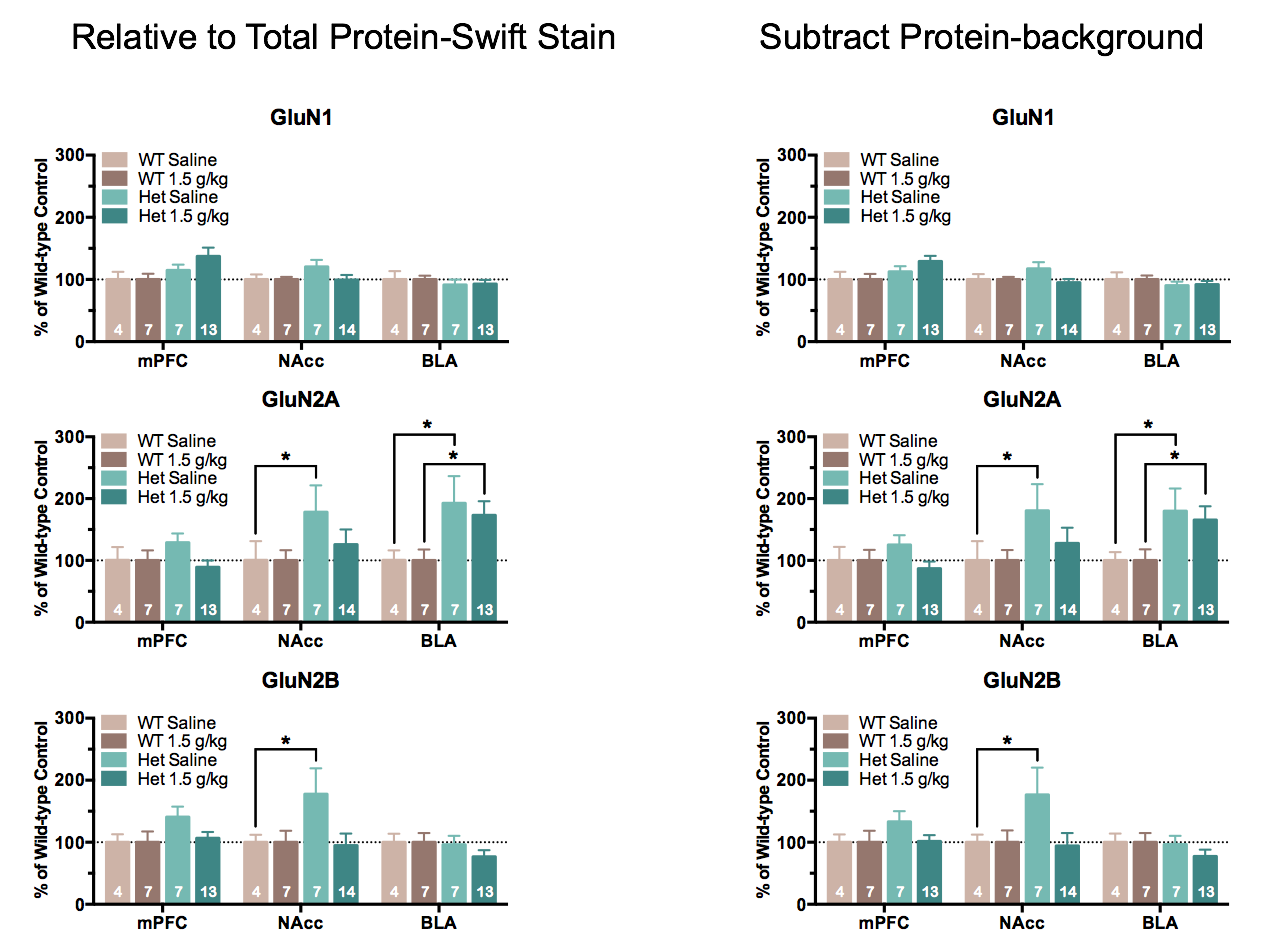

Supplement: Supplementary file 1 [file DataSheet1.DOCX]
